# Vertical Banded Gastroplasty Revisions: A Single-Center Experience

**DOI:** 10.7759/cureus.94559

**Published:** 2025-10-14

**Authors:** Haider Alshurafa, Faisal S AlGhamdi, Fatmah Alrawaji, Hassan Alshurafa, Shaden AlGhamdi, Bassam Alhassan, Khaled Altoukhi

**Affiliations:** 1 Hepatobiliary, Bariatric and General Surgery, Prince Sultan Military Medical City, Riyadh, SAU; 2 General Surgery, Prince Mohammed Bin Abdulaziz Hospital, Riyadh, SAU; 3 General Surgery, King Saud Medical City, Riyadh, SAU; 4 Medical School, King Saud University Medical City, Riyadh, SAU; 5 General Surgery, Prince Sultan Military Medical City, Riyadh, SAU; 6 Surgical Oncology, Upper Gastrointestinal and General Surgery, Prince Sultan Military Medical City, Riyadh, SAU

**Keywords:** bariatric surgery, obesity, revisional surgery, roux-en-y gastric bypass, vbg, vbg revision, vertical banded gastroplasty

## Abstract

Background

Revising bariatric procedures presents significant challenges, particularly in patients with previous vertical banded gastroplasty (VBG). Among the various revision techniques, Roux-en-Y gastric bypass (RYGB) and VBG reversal are the most commonly performed.

Methodology

This retrospective study analyzed all patients who underwent revision after VBG at our center between 2010 and 2021. Data were reviewed regarding indications, preoperative planning, surgical techniques, and early outcomes.

Results

Of the 234 revisional bariatric procedures performed between 2010 and 2021, 55 (23.5%) were VBG revisions. Among these, 33 patients underwent conversion to RYGB, 16 had gastrogastrostomy-assisted VBG reversal, three underwent mini-gastric bypass, and one each underwent conversion to laparoscopic sleeve gastrectomy, biliopancreatic diversion, and anterior plication. All but three procedures were completed laparoscopically. No mortalities occurred. Postoperative complications included two anastomotic leaks and two cases of intraluminal bleeding. The most common indication for reversal was excessive weight loss, whereas inadequate weight loss was the leading indication for conversion.

Conclusions

VBG revision is a technically demanding procedure that requires advanced surgical expertise. Laparoscopic revision is a safe, practical, and effective solution for VBG failure or complications.

## Introduction

Due to the fast rise in the prevalence of morbid obesity, it has grown into a significant public health concern [1]. Globally, more than 1.7 billion people are affected [2]. Currently, the most effective long-term treatment for obesity is bariatric surgery, which enhances patients’ quality of life [3]. As the number of bariatric surgeries increases, patients needing revisional procedures are increasing too [4]. Vertical banded gastroplasty (VBG) was a popular restrictive bariatric procedure in the 1990s, but it is no longer used in bariatric surgery because of its late complications and insufficient late weight loss [5]. Several studies [6-8] have reported that 25-54% of VBG patients required revisional surgery. Revising VBG patients with other bariatric operations has been reported involving several techniques, including the most common ones, such as the conversion to Roux-en-Y gastric bypass (RYGB) [9,10], laparoscopic sleeve gastrectomy (LSG) [11,12], mini-gastric bypass (MGB) [13,14], biliopancreatic diversion (BPD) [15,16], and VBG reversal [2,17].

The revision of the VBG depends on the experience of the surgical team, their commonly practiced procedures, and the reason for the revision, including weight gain, which persists in a high proportion of VBG patients [18], long-term gastric outlet obstruction, and reflux esophagitis. Vomiting and/or dysphagia that cannot be controlled by medical treatment requires surgical revision. Laparoscopic conversion to RYGB is the most preferred revisional procedure for failed VBG [19-24], but there are other conversion procedures reported in the literature with variable outcomes [5,9]. Research has been performed to assess the success of one or two revisional surgeries following a failed VBG. In this study, whether in terms of weight loss or consequences from VBG, we assessed a wide range of revisional techniques for failed VBG, with the objective of determining the safety and effectiveness of RYGB compared to other revisional procedures after failed VBG.

## Materials and methods

A retrospective review of all the patients’ medical records who underwent additional bariatric surgeries following VBG was conducted between August 2010 and November 2021. Our inclusion criteria were all patients with prior VBG who underwent revisional bariatric surgery, regardless of whether the first procedure was open or laparoscopic, performed at our facility or somewhere else. After obtaining institutional review board approval from our facility, we collected and analyzed data from the preoperative, surgical, and postoperative stages.

The indications for the revisions, type of revision procedure, surgical techniques of the revisions, complications, and immediate outcomes were also analyzed. Failure to lose weight was defined as a body mass index (BMI) of more than 35 kg/m^2^ or weight loss of less than 50% of excess body weight without patient satisfaction. Massive weight loss with a BMI of less than 18 kg/m^2^ was another reason for revision, usually due to technically excessive surgical restriction or due to decreased food intake in consequence to gastroesophageal reflux disease (GERD) or persistent vomiting. The complications of VBG include recurrent admissions with vomiting and dehydration, stomal obstruction, and GERD.

Before the revisional procedures, all patients were assessed and underwent a physical examination, followed by full laboratory investigations with deficiencies corrected. All patients were evaluated by preoperative upper gastrointestinal (GI) endoscopy and an upper GI contrast study. Any patient who tested positive for *Helicobacter pylori* was treated preoperatively. A preoperative dietary and psychological evaluation was done for all patients. The decision for the revisional procedure was taken by the multidisciplinary team upon patient approval. After data collection, continuous variables were reported as mean values with ranges, and categorical variables were presented as frequencies and percentages. Data are summarized as text and tables to illustrate the distribution of revisional procedures and patient characteristics, while outcomes and complications are described descriptively without formal statistical testing, to share our experience case-by-case, specifically for the reading surgeons facing similar cases.

Surgical procedures

All procedures were started laparoscopically, under general anesthesia, in the supine reverse Trendelenburg position, with preoperative antithrombotic and antibiotic prophylaxis. A Veress needle was used to create the pneumoperitoneum, and procedures started with adhesiolysis and an assessment of the feasibility of the revisional procedure.

Laparoscopic Revisional Roux-en-Y Gastric Bypass

A gastric pouch was created by transecting the stomach horizontally over the location of the former stomal mesh, where the tissues appeared healthy. The excluded fundus, remnant VBG pouch with staple lines, and mesh stoma were all resected together. The vascularity of the new gastric pouch was secured by preserving the left gastric artery. Thick stapler size (green or black reload) was used in most cases in the creation of the gastric pouch, depending on the scarring and tissue thickness. The staple lines on both sides, as well as the new gastric pouch and excluded stomach, were invaginated continuously with absorbable monofilament 3/0 sutures. A 30 mm linear stapler (blue or purple reload) was used to create the gastrojejunal anastomosis with the jejunum at 50-60 cm from the ligament of Trietz. The anticolic and antigastrically placed alimentary limb measured 120-200 cm, depending on the patient’s BMI. The jejunojejunal anastomosis was made with a 60 mm linear stapler (white or tan reload), and the jejunostomy was closed with two layers of 3/0 PDS running suture. The Peterson and mesenteric defects were never opened.

Laparoscopic Reversal of Vertical Banded Gastroplasty

A gastrotomy is typically created distally on the healthy section of the stomach to relieve limitation at the narrowing stoma. Thick staples (green or black in color) were then introduced through the stoma with the aid of the orogastric tube and fundus. Another needle of the same size was used to create a wide gastro-gastric stoma. The staple line was then checked for bleeding before the completion of the operation and closure of the gastrostomy.

Laparoscopic Conversion to Mini-Gastric Bypass

A gastrostomy was typically performed distally, with the stapling device inserted through the stoma with the guide of the orogastric tube and fundus, and used to remove restriction at the narrowed stoma and complete gastro-gastric anastomosis until the end of the fundus. The distal end of the gastric pouch was created at the incisura level using thick staples transversely. Subsequently, the longitudinal pouch was created over the 38-French orogastric calibration tube. The lateral part of the excluded stomach was usually removed, including the gastrostomy site. With absorbable monofilament 3/0 sutures, the staple lines on both sides, the new gastric pouch, and the excluded stomach were invaginated continuously. Depending on the patient’s BMI, the gastro-jejunal anastomosis was made with a 30 mm linear stapler (blue or purple refill) 150-200 cm from the ligament of Trietz.

Laparoscopic Conversion to Sleeve Gastrectomy

The silicone ring was first taken off. To relieve the limitation at the narrower stoma, a gastrotomy was performed distally on the stomach, and using strong staples (green or black), the stapling device was introduced through the stoma and fundus. The more curved vessels were separated. A 38-French orogastric tube was used for the sleeve gastrectomy. A 60 mm black round was shot into the antral staple line first, 2-4 cm from the pylorus, followed by four to five reloads of 60 mm (blue or purple reloads). Utilizing 3/0 PDS running sutures together with an antropexy and omental patch, the staple line was invaded.

Laparoscopic Conversion to Biliopancreatic Diversion

A gastrostomy was made distally at the site where the distal part of the stomach would be excised, and using thick staples (green or black reload), the stapling device was inserted through the stoma with the guide of the orogastric tube and fundus, followed by completion of the gastro-gastric anastomosis till the end of the fundus. The stomach was transected at the midpoint of the lesser curvature to create a gastric pouch of around 200 mL volume.

Laparoscopic Plication of the Vertical Banded Gastroplasty Pouch

Extensive adhesiolysis was done. The lesser sac could not be approached. Using a continuous suture method, the anterior wall of the VBG pouch was plicated in two layers with Prolene 3/0.

Postoperative course

For the first 24 hours, the patients were kept with no oral intake. Then, they underwent a gastroscopy to evaluate the procedure and rule out any leaks or obstructions. The patients were allowed to gradually begin oral consumption after that. All patients were administered a prophylactic antithrombotic with enoxaparin, a prophylactic antibiotic, with proton pump inhibitors, analgesia, intravenous fluids, and an antiemetic. Early mobilization with an incentive spirometer was practiced for all patients. After discharge, the patients were regularly followed up in the outpatient clinic.

## Results

General demographic data

Over 10 years, between August 2010 to November 2021, 2,463 bariatric procedures were performed, with 234 revisional procedures (9.5% of all the bariatric procedures). Of these 234 revisions, there were 55 (23.5%) VBG revisions. The mean age of the patients was 41.1 years, ranging between 21 and 63 years old. The majority of patients were female (74%). The patients’ weight ranged between 55 and 180 kg, with an average of 95.89 kg. Height was between 135 and 187 cm, with an average of 161.64 cm. The mean BMI was 41.45 kg/m^2^, with a range between 17.4 and 73 kg/m^2^. The distributions of the revisional procedures after VBG are presented in Table 1. All VBG cases were found with Prolene mesh, except for three patients with Proring band, where there was more stenosis. VBG revision procedures were done laparoscopically, with gastrogastrostomy performed two to three times. The gastroplasty in VBG was converted because of severe GERD, recurring blockage and stenosis, malnutrition, atonia of the pouch, and insufficient weight gain associated with staple line rupture.

**Table 1:** Distribution of revision procedures after VBG VBG = vertical banded gastroplasty; RYGB = Roux-en-Y gastric bypass; MBG = mini-gastric bypass; BPD = biliopancreatic diversion; LSG = laparoscopic sleeve gastrectomy

Revisional procedure	Number of patients	Percentage (%)
RYGB	33	60.5
VBG reversal	16	28.5
MGB	3	5.6
BPD	1	1.8
LSG	1	1.8
Anterior plication	1	1.8
Total	55	100%

Procedure-specific demographic data

The conversion to RYGB procedures was laparoscopic, with the exception of two cases that were converted to open as both were post-open VBG, as they were in the initial experience. Regarding age, patients who underwent conversion to BPD, LSG, and anterior plication had a mean age of 39 years, 39 years, and 57 years, respectively. However, the average was 41.3 years for RYGB and 40.6 years for MGB reversal. Before surgery, patients who underwent RYGB conversions had significantly higher average BMIs (43.8 kg/m^2^) than those who chose VBG revision (31.4 kg/m^2^). Average BMI for MGB reversal was 35 kg/m2, while for the three patients with BPD reversal, LSG reversal, and anterior plication, the average BMI was 57.5 kg/m^2^, 35.9 kg/m^2^, and 46 kg/m^2^, respectively. The patients who underwent conversion to RYGB were older and had a higher BMI, mainly due to weight regain with GERD.

Conversion to BPD was performed on a 39-year-old female with polio and a BMI of 57.7 kg/m^2^. This patient was seven years post-open VBG with massive adhesions at the angle of His. The procedure included reversal of VBG and BPD with a gastric pouch volume of around 200 mL, and the diversion was created using a 150 cm common channel. The patient had an uneventful postoperative course associated with a very good weight loss. Reversal LSG was performed on a 39-year-old female with a BMI of 39.5 kg/m^2^. The patient has a nine-year-old laparoscopic VBG with a large gastric pouch, a large gastric stoma, and minimal adhesions. The patient underwent an uneventful LSG with no drain and a satisfactory weight loss progression.

Anterior plication resection was done on a 57-year-old female with a BMI of 46 kg/m^2^. The patient had a post-open VBG with extensive adhesions posterior to the stomach, VBG leak, chronic pancreatitis, a large gastric pouch, and a failure to lose weight. Gastric pouch plication was the only viable option, with an uneventful postoperative course.

Complications

Five patients who underwent the VBG revision procedure required further bariatric operations because of weight regain (three underwent conversion to RYGB, and two patients underwent conversion to MGB). For the two MGB patients, both had leaks, one treated by stent and drainage, and the other treated by conversion to RYGB. Overall, three patients were revised by MGB, including the two patients post-revision of VBG. A 47-year-old female patient with post-open VBG underwent MGB directly as the primary conversion procedure. A 35-year-old female patient post-revision of VBG underwent MGB due to weight regain and development of a gastro-pleural fistula after MGB for one year. The patient was treated by laparoscopic exploration and conversion of MGB to RYGB with a mini-thoracotomy to excise the fistula. A 40-year-old male patient post-revision of VBG with weight regain underwent MGB and developed a leak post-MGB. The patient was treated by laparoscopic drainage and a stent for eight weeks and had complete resolution. All three patients had satisfactory weight loss subsequently.

Patients who underwent laparoscopic procedures had shorter operating times compared to those who underwent open RYGB conversion. A 28-year-old male with a BMI of 49.5 kg/m^2^ presented on day three post-laparoscopic conversion to RYGB with a radiologically confirmed leak, undergoing open exploration with drainage of the collection and repair of the leaking area. This patient had a postoperative incisional hernia, but he lost satisfactory weight. For the RYGB procedure, one patient was investigated after one week because of abdominal pain, vomiting, and a query leak, but was excluded. While two patients were revised after five and seven years because of weight regain by resizing the gastric pouch, refashioning the gastrojejunal anastomosis, and lengthening the alimentary limb. One of the patients who underwent conversion to RYGB had a leak, which was treated with drainage and a nasojejunal feeding tube. Two patients with weight regain who underwent conversion to RYGB had gastro-gastric fistulas and underwent successful laparoscopic revisional RYGB with satisfying postoperative weight loss. Most patients undergoing conversion to RYGB had drains, and one patient had a combined procedure with ventral hernia repair. One case of conversion to RYGB combined with posterior cruroplasty due to a large hiatus hernia. This patient had a drop in hemoglobin due to intraluminal bleeding, which was managed conservatively.

## Discussion

Dr. Edward E. Mason of the University of Iowa first suggested open VBG in 1980 as a substitute for RYGB [25]. On long-term follow-up, VBG was linked to a significant demand for revisional surgery. Short-term results were very encouraging; however, sustained weight loss over the long term was difficult, and two decades later, initial enthusiasm for this method had waned [7,26]. Revisional surgery following VBG occurs in 10-56% of cases, according to research [7,8]. RYGB demonstrated the most positive prospects regarding weight loss, quality of life, and satisfaction, even though other conversion types, including those performed in this study (MGB, BPD, LSG, and anterior plication), have been advocated over the years. A revision VBG was mostly suggested in early studies; however, this was incredibly unsuccessful and increased the likelihood of problems requiring re-intervention in up to 68% of cases [7,8,21]. Alternative procedures include band-on VBG, duodenal switch, or even conversion to sleeve gastrectomy.

Due to stretchable intraperitoneal adhesions, particularly those around the stomach, revisional bariatric surgery is frequently difficult and technically complex. A laparoscopic method is preferred if possible. With fundectomy, the approach is typically lateral. Roux limb is typically 150-200 cm, gastro-jejunostomy anastomosis is typically 30 mm (except in the first five cases, where our method for gastro-jejunostomy anastomosis used a transoral circular stapler), and above the banding mesh or Proring site. The two main causes of revision surgery after open VBG are outlet obstruction (stoma stenosis) with significant weight loss and recurrent weight gain (due to staple line rupture). As a result, assessing weight loss alone is not a helpful measure to assess whether VBG is effective; rather, it can be used in conjunction with other factors such as quality of life, nutritional anamnesis, and the patient’s perception of weight loss. In the investigations, the prevalence of staple line rupture was up to 48% [27]. Although endoscopic dilatation and the simple removal of the Dacron/Marlex mesh were suggested for stoma stenosis, the results were not satisfactory over the long term [28].

In general, more issues have been reported following conversion surgery [26]. Nevertheless, in our view, converting open VBG to RYGB is secure and efficient. Except for two cases of leaks, we had no major problems with our series. Numerous studies in the literature recommend transecting the original vertical staple line due to the possibility of an ischemic zone developing between the original and new staple lines [4,26].

Conversion to RYGB is always the preferred treatment for revision of VBG for weight regain due to issues with revisional MGB. BPD reversal includes reversal of VBG, BPD, and diversion made using a 150 cm common channel, all of which were reversed using a gastric pouch volume of about 200 mL. The patients lost a large amount of weight and had an uneventful postoperative course.

The limitations of the study include the small sample size. Further, as it was a retrospective study, there were some limitations in the data collection, which included some missing data, especially at that time, the patient files were paper-based. Additionally, the less frequent revisional procedures, such as anterior plication, LSG, BPD, and MGB, were identified as per patient condition as detailed previously. Hence, further studies are warranted to confirm these findings.

## Conclusions

According to our observations, the recommended course of action for treating failed VBG for weight regain is conversion to RYGB. Patients who have already undergone VBG may develop problems, such as gaining weight several years after the initial treatment. Revisional procedures could be performed using a laparoscopic technique in most cases. Some patients gain weight after revision of VBG, and many return to being morbidly obese. In the majority of patients, conversion to RYGB is linked to weight reduction and the resolution of severe obesity, in addition to the resolution of GERD symptoms. Hence, it is our recommended procedure for conversion after VBG.
